# Ultrasound-Assisted Deep Eutectic Solvent Extraction of Flavonoids from *Hovenia dulcis* Thunb: Optimization, Phytochemical Profiling, and Antioxidant and Anti-Inflammatory Activity Evaluation

**DOI:** 10.3390/molecules31193425

**Published:** 2026-09-26

**Authors:** Lei Zhou, Haixin Li, Xu Zhou, Xueying Zou, Guanfeng He

**Affiliations:** School of Pharmacy, Guangdong Pharmaceutical University, Guangzhou 510006, China

**Keywords:** *Hovenia dulcis* Thunb, deep eutectic solvents, flavonoids, antioxidant, anti-inflammatory, UPLC-Q-Exactive Orbitrap-HRMS

## Abstract

*Hovenia dulcis* Thunb. is a traditional medicinal and edible plant that is widely recognized for its hepatoprotective and hangover-alleviating properties, which are largely attributed to its rich flavonoid composition. The aim of this study was to develop an environmentally friendly ultrasonic-assisted deep eutectic solvent (UAE-DES) extraction method for the efficient extraction of bioactive flavonoids from *Hovenia dulcis* Thunb. A Box–Behnken design (BBD) was employed to systematically optimize the extraction parameters. Under optimal conditions (water content of 36%, liquid-to-solid ratio of 42:1 mL/g, extraction time of 42 min), the extraction yield reached a maximum of 9.09 ± 0.04%. Following purification using AB-8 macroporous resin, the phytochemicals were comprehensively characterized by UPLC-Q-Exactive Orbitrap-HRMS. A total of 12 flavonoids were identified. Bioassays demonstrated that the purified flavonoids exhibited strong, dose-dependent antioxidant activity against DPPH and ABTS radicals in vitro. Furthermore, the extract demonstrated significant anti-inflammatory activity by markedly inhibiting the excessive production of nitric oxide (NO) in RAW 264.7 macrophages induced by lipopolysaccharide (LPS), whilst exhibiting no cytotoxicity within the tested concentration range (0.06–0.96 mg/mL). These findings strongly confirm that the optimized UAE-DES method is a sustainable and efficient strategy for extracting structurally intact and pharmacologically active flavonoids from *Hovenia dulcis* Thunb, highlighting its immense potential for future applications in the functional food and pharmaceutical industries.

## 1. Introduction

*Hovenia dulcis* Thunb., Rhamnaceae (*H. dulcis*) is a hardwood tree species belonging to the genus *Hovenia* in the family Rhamnaceae, which is characterized by swollen, edible fruit stalks. It is a well-known traditional medicinal and edible plant native to East Asia [1]. Traditionally, this substance has been widely used for its remarkable efficacy in alleviating hangovers. Recent pharmacological studies have extensively demonstrated its significant therapeutic role in the treatment of alcohol-related liver disease and in enhancing alcohol metabolism [2,3,4,5]. *H. dulcis* extracts also offer a wide range of health benefits, including alleviating hyperglycemia and complications of diabetes [6,7,8], improving liver fibrosis [9], and regulating gut dysbiosis and fatty acid metabolism in the liver [10]. These diverse biological activities are fundamentally attributable to their high content of naturally occurring bioactive macromolecules and secondary metabolites, particularly polysaccharides and flavonoids [9,11].

Among these phytochemicals, flavonoids such as dihydroquercetin and total seed flavonoids have attracted widespread attention as the key active constituents responsible for the plant’s potent antioxidant, anti-diabetic and liver-protective properties [6,9]. Although the flavonoids found in *H. dulcis* possess significant pharmacological potential and high added value, the development of efficient and environmentally friendly extraction methods remains a key problem to their industrial application. Traditional extraction techniques rely primarily on hot water or volatile organic solvents; however, these methods often have inherent limitations, such as low extraction yields, lengthy processing times, potential environmental toxicity, and the thermal degradation of sensitive bioactive compounds [12,13]. Therefore, there is an urgent need to develop novel, environmentally friendly, and efficient extraction methods. Exploring sustainable alternatives is not only crucial for maximizing extraction efficiency and preserving the bioactivity of *H. dulcis* flavonoids, but has far-reaching implications for promoting the high-value utilization and environmentally friendly processing of this traditional medicinal resource.

Recently, deep eutectic solvents (DESs) have emerged as highly promising alternatives to traditional organic solvents [14]. DESs possess an extremely low vapor pressure, excellent biodegradability, tunable polarity, and extensive hydrogen-bonding networks, and exhibit outstanding solubilization capabilities for phenolic and flavonoid compounds [15,16]. As the cavitation and mechanical shear forces generated by ultrasound effectively disrupt plant cell walls and reduce the high viscosity inherent in DESs, combining DESs with ultrasound-assisted extraction (UAE) produces a powerful synergistic effect, thereby significantly accelerating mass transfer rates and improving extraction efficiency [17,18]. Crucially, DESs create a unique protective microenvironment that shields sensitive flavonoids from thermal or oxidative degradation during the extraction process, thereby maximizing the retention of their inherent biological and antioxidant activities [19,20]. Although these significant advantages have been demonstrated in various plant substrates, to the best of our knowledge, there have been no reports on the use of an ultrasound-assisted deep eutectic solvent extraction (UAE-DES) strategy to extract functional flavonoids from *H. dulcis*. Therefore, this study aims to identify the optimal DES system for *H. dulcis* flavonoids and systematically optimize the key extraction variables utilizing a Box–Behnken design (BBD). The crude extracts were subsequently enriched using AB-8 macroporous resin, profiled via UPLC-Q-Exactive Orbitrap-HRMS, and evaluated for their in vitro antioxidant and anti-inflammatory activities. This research seeks to provide a sustainable and efficient model for the high-value utilization of this functional plant resource.

## 2. Results and Discussion

### 2.1. Screening of the Optimal DES System

As shown in Table 1, twelve distinct ChCl-based DES formulations incorporating four representative HBDs (urea, glycerol, 1,4-butanediol, and malonic acid) at varying molar ratios (1:1, 1:2, and 1:3) were systematically screened to evaluate their capacity for extracting flavonoids from *H. dulcis*. During this initial screening phase, the preliminary extraction parameters were held constant: an ultrasonic time of 30 min, an extraction temperature of 45 °C, an ultrasonic power of 300 W, a DES water content of 30%, and a liquid-to-solid ratio of 5:1 mL/g. As illustrated in Figure 1, the extraction yields of total flavonoids varied dramatically across the different DES formulations and the 70% ethanol control. Notably, DES-10 (ChCl-MA 1:1) exhibited the highest extraction efficiency among all tested solvents (labeled with ‘a’). Although the yield obtained using DES-11 (ChCl-MA 1:2, labeled with ‘ab’) showed no statistically significant difference from that of DES-10 (*p* > 0.05), DES-10 consistently demonstrated a marginally superior and more stable extraction yield. Meanwhile, the other DES formulations yielded moderate extraction efficiencies, distributing across the intermediate statistical tiers (‘bc’, ‘cd’, and ‘d’). Conversely, the conventional 70% ethanol solvent displayed the lowest recovery rate, falling into the bottom statistical tier (‘e’). These significant disparities underscore the exceptional solubilization capacity and matrix-disrupting ability of the DES-10 system. Consequently, supported by its statistical superiority, DES-10 was selected as the optimal solvent for all subsequent optimization experiments.

This prominent extraction superiority can be rationalized by multiple synergistic mechanisms. Structurally, flavonoids from *H. dulcis* (such as dihydromyricetin and rutin) possess abundant phenolic hydroxyl groups, which can act as potent hydrogen-bond donors or acceptors. Malonic acid, as a dicarboxylic acid, offers strong hydrogen-bonding acidity and a high density of carboxyl groups. This matches perfectly with the molecular structure of *H. dulcis* flavonoids, facilitating the formation of an extensive and stable intermolecular hydrogen-bonding network between the solvent and the solute molecules. Furthermore, the molar ratio of HBA to HBD significantly influences the thermodynamic properties and steric hindrance within the solvent matrix. At a 1:1 molar ratio, the ChCl-MA system likely achieves an optimal balance between moderate polarity and favorable molecular interactions. While higher ratios of HBD (such as 1:2 or 1:3) might theoretically increase hydrogen-bonding sites, they often trigger an increase in system viscosity or alter the spatial arrangement, which hampers acoustic cavitation during ultrasound processing and restricts mass transfer. From a physical chemistry perspective, the remarkable extraction efficiency of this UAE-DES system is governed by the synergistic interplay between solvent functionalization and mechanical cell wall disruption. As demonstrated by recent studies [21,22], organic acid-based DESs act as aggressive hydrogen-bond donors (HBDs) that actively compete with and dissociate the dense intermolecular hydrogen-bonding networks within the plant cellulose and flavonoid-lignin matrices. Simultaneously, the propagation of ultrasonic waves generates localized transient acoustic cavitation bubbles. The violent collapse of these bubbles produces high-speed microjets and shock waves that shatter the cellular structures of *H. dulcis* [21]. This intense mechanical disruption not only decreases the interfacial tension but also dramatically accelerates the intracellular mass transfer of target flavonoids into the eutectic solvent matrix, thereby explaining the significantly higher yield compared to conventional organic solvents [23]. Consequently, ChCl-MA (1:1) was selected as the optimal extraction solvent for all subsequent optimization experiments.

### 2.2. Single-Factor Experiments on the Extraction Yield of Flavonoids

#### 2.2.1. Effect of Water Content in DES

Pure DESs typically suffer from high viscosity, which impedes efficient mass transfer during extraction. Adding an appropriate amount of water can overcome this barrier. According to Figure 2A, the extraction yield climbed rapidly as the water content increased from 5% to 20%, maintaining a high plateau up to 40% (reaching a peak of approximately 7.89%). This highlights that moderate hydration dramatically lowers the viscosity and improves the fluidity of the DES, expediting flavonoid extraction. Conversely, when the water content exceeded 40%, the yield dropped precipitously (falling to 1.24% at 100% water content). This drastic decline occurs because excessive water disrupts the extensive intermolecular hydrogen-bonding network between the hydrogen-bond acceptor and donor within the DES [24]. Furthermore, excessive water shifts the solvent polarity, rendering it less favorable for the solubilization of lipophilic flavonoids.

#### 2.2.2. Effect of Liquid-to-Solid Ratio

The liquid-to-solid ratio determines the concentration gradient between the plant interior and the bulk solvent, which acts as the primary driving force for mass transfer. As shown in Figure 2B, the extraction yield displayed a distinct “rise-and-fall” pattern. As the ratio increased from 5:1 to 40:1 mL/g, the yield surged to a maximum of 10.19%. This initial improvement occurs because a larger solvent volume expands the contact area with the plant matrix, accelerating the release of flavonoids. However, when the ratio exceeded 40:1 mL/g, the yield experienced a noticeable decline. This reduction is likely due to the excessive solvent volume, which not only over-dilutes the target flavonoid concentration but also attenuates the mechanical and cavitational energy of the ultrasound waves, thereby reducing the mass transfer efficiency [25]. Additionally, an overabundance of solvent promotes the dissolution of unwanted impurities, complicating subsequent purification and increasing operational costs.

#### 2.2.3. Effect of Extraction Temperature

The extraction temperature is a critical thermodynamic parameter that significantly influences solvent viscosity and molecular diffusion. As illustrated in Figure 2C, the extraction yield of total flavonoids exhibited a continuous and significant upward trend as the temperature increased from 25 °C to 65 °C. Between 50 °C and 65 °C, the yield reached a stable maximum with no statistical difference across this range (all labeled with ‘a’). Although the yield stabilized at higher temperatures, temperatures exceeding 65 °C were not investigated. Excessive heat increases solvent vapor pressure, which dampens ultrasonic cavitation intensity and may induce the thermal degradation of heat-sensitive flavonoids such as dihydromyricetin. Accordingly, 60 °C was selected for subsequent extractions to balance energy efficiency and maximum yield. This phenomenon can be mainly attributed to the fact that elevated temperatures effectively reduce the inherent high viscosity of the deep eutectic solvent (DES). The decreased viscosity significantly enhances the thermal motion of molecules and improves the permeability of the solvent into the *H. dulcis* cellular matrix. Consequently, the diffusion coefficient between the solute and the solvent is magnified, thereby facilitating the rapid dissolution of flavonoids.

#### 2.2.4. Effect of Extraction Time and Ultrasonic Power

The duration and intensity of the ultrasonic treatment are vital for efficient cell wall disruption. As depicted in Figure 2D, the extraction yield increased steadily during the initial 40 min, reaching a peak of 6.11%, after which the curve plateaued and slightly declined. This indicates that the flavonoids had achieved dissolution equilibrium; prolonged ultrasonic exposure does not further improve the yield and may even induce structural degradation of the extracted bioactive compounds. Meanwhile, as shown in Figure 2E, the extraction yield plateaued rapidly between 300 W, 400 W, and 500 W, displaying no significant difference across this high-power range (all labeled with ‘a’, *p* > 0.05), while 100 W yielded the lowest extraction yield. Therefore, 400 W was selected for subsequent experiments as an adequate power level to ensure sufficient cavitation without wasting energy.

#### 2.2.5. Selection of Variables for Response Surface Optimization

Based on the comprehensive analysis of the single-factor experiments, ultrasonic power and extraction temperature were set at their optimal levels (400 W and 60°C, respectively) for subsequent experiments. Meanwhile, water content, liquid-to-solid ratio, and extraction time were identified as the most influential parameters with clear optimal peaks. These preliminary single-factor screenings were essential to narrow down the parameter ranges, identify the central points (0 level) for the Box–Behnken Design (BBD), and fix less critical factors to minimize unnecessary multidimensional experimental runs. Consequently, to further maximize the extraction efficiency and explore their interactive effects, these three variables were selected for BBD optimization. The independent variables were symmetrically coded at three levels based on their respective optimal single-factor ranges: 20%, 40%, and 60% for water content; 20:1, 40:1, and 60:1 mL/g for liquid-to-solid ratio; and 20, 40, and 60 min for extraction time.

### 2.3. Box–Behnken Design (BBD) Optimization

#### 2.3.1. Statistical Analysis and Model Fitting

As shown in Table 2, a three-factor (water content (A), liquid-to-solid ratio (B), and extraction time (C)), three-level (A: 20, 40, 60%; B: 20, 40, 60 mL/g; C: 20, 40, 60 min) Box–Behnken experimental design was used. A second-order polynomial equation was fitted to the experimental data, and the resulting quadratic model was subjected to ANOVA, with the results compiled in Table 3. The final predictive empirical model obtained is given by the following equation: Y = 9.11 − 0.1800A + 0.0950B + 0.1550C − 0.3050AB + 0.2050AC − 0.3900BC − 0.4680A^2 − 0.5580B^2 − 0.3830C^2. The model proved highly significant, as reflected by its F-value of 138.01 and P-value below 0.0001. The lack-of-fit test yielded a *p* of 0.2229 (>0.05), indicating no significant deviation from the pure error and confirming that the model adequately captured the experimental trends. The coefficient of determination (R^2^ = 0.9944) and its adjusted counterpart (*Adj* R^2^ = 0.9872) together indicated that more than 99.4% of the response variation could be explained by the model. The predicted R^2^ (*Pred* R^2^ = 0.9403) agreed closely with the adjusted R^2^ (difference <0.2), highlighting strong predictive reliability. A low coefficient of variation (C.V. = 0.74%) further attested to the precision and reproducibility of the measurements. The Adeq Precision value, a signal-to-noise indicator, reached 33.00, far exceeding the recommended minimum of 4.0, demonstrating that the model provided sufficient resolution to navigate the design space and achieve reliable optimization.

#### 2.3.2. Effect of Independent Variables and Their Interactions

The significance of each model term was evaluated based on their respective *p*-values. As shown in Table 3, all the linear terms (A, B, C) and quadratic terms (A^2^, B^2^, C^2^) were highly significant (*p* < 0.01), indicating that water content, liquid-to-solid ratio, and time independently and non-linearly affected the flavonoid yield. More crucially, all three interaction terms—water content and liquid-to-solid ratio (AB, *p* < 0.0001), water content and extraction time (AC, *p* = 0.0003), and liquid-to-solid ratio and extraction time (BC, *p* < 0.0001)—exhibited highly significant interactive effects. To visually elucidate these interactions, three-dimensional (3D) response surface plots and their corresponding 2D contour plots were constructed (Figure 3). The steep nature of the 3D surfaces vividly illustrated the strong synergistic effects among the variables. For instance, the significant interaction between water content and liquid-to-solid ratio (AB) can be attributed to the physicochemical dynamics of the DES. An optimal addition of water significantly reduces the inherent viscosity of the DES, which, when coupled with an appropriate liquid-to-solid ratio, maximizes the concentration gradient and mass transfer efficiency. However, concurrently exceeding the optimal limits of both parameters would over-dilute the solvent system, severely weakening the hydrogen-bonding network and the acoustic cavitation intensity, thereby causing a sharp decline in extraction yield. Similarly, the significant BC interaction suggests that a larger solvent volume requires a precisely matched ultrasonic duration to ensure uniform energy dissipation; excessive time and solvent volume merely waste energy and may induce the structural degradation of the extracted flavonoids.

#### 2.3.3. Optimization and Model Validation

Based on the numerical optimization function of the Design-Expert software, the optimal extraction conditions to maximize the total flavonoid yield were predicted to be a water content of 35.816%, a liquid-to-solid ratio of 42.227 mL/g, and an ultrasonic extraction time of 41.789 min. Under these theoretically optimal conditions, the model predicted a maximum extraction yield of 9.137%. To validate the reliability and accuracy of the predictive model, triplicate verification experiments were conducted under the suggested optimal conditions (adjusted slightly to 36%, 42 mL/g, 42 min for practical operability). The actual experimental yield obtained was 9.09 ± 0.04%, which was in good agreement with the predicted value. This high concordance confirms that the optimized UAE-DES process is robust, reliable, and highly efficient for the extraction of flavonoids from *H. dulcis*.

### 2.4. Chemical Composition Analysis of the Purified Flavonoids

An AB-8 macroporous resin column was employed to effectively desorb and enrich the flavonoids. The application of macroporous resin successfully eliminated the DES matrix, ensuring that the subsequent bioactivity evaluations reflect the true pharmacological potential of the purified *H. dulcis* flavonoids.

The phytochemical profile was characterized using UPLC-Q-Exactive Orbitrap-HRMS. Identification of the constituents relied on comparing the observed MS^1^ and MS^2^ spectra with self-built database, leading to the tentatively identified of 12 distinct flavonoids. Their chromatographic profile, acquired in negative ionization mode, is presented as the total ion chromatogram in Figure 4, with detailed identification parameters (including retention times, molecular ions, and fragment ions) provided in Table 4. The chemical structures of these annotated constituents are illustrated in Supplementary Figure S1, providing a clear visual reference for their structural classifications. Among them, the identified constituents are predominantly flavonoids and their derivatives, which can be further classified into specific subcategories. Specifically, three flavanonols (dihydromyricetin, dihydroquercetin and pinobanksin), five flavonols (myricetin, quercetin, kaempferol, galangin and kaempferide), one flavonone (pinocembrin), one dihydrochalcone (izalpinin) and two flavones (parvisoflavone B and atalantoflavone) were identified. The comprehensive identification of these specific flavonoids is strongly corroborated by established chemotaxonomic profiles and a phytochemical study of *H. dulcis* [26,27]. Quantitative chromatographic investigations by Park et al. [28] similarly demonstrated that these four core flavonoids dominate the chemical landscape of *H. dulcis* fruit extracts, which aligns perfectly with our HRMS profile.

### 2.5. ABTS and DPPH Radical Scavenging Capabilities

The in vitro antioxidant activities of the purified flavonoids were systematically evaluated using ABTS and DPPH radical scavenging assays. Trolox was utilized as the positive reference standard. As illustrated in Figure 5, the purified flavonoid extract exhibited a dose-dependent radical scavenging capacity in both assays. In the ABTS assay, the extract demonstrated an IC_50_ value of approximately 2.61 mg/mL and a maximum clearance rate of 87.0%, an antioxidant capacity equivalent to 1.74 mM Trolox. In the DPPH assay, the IC_50_ was calculated to be approximately 1.23 mg/mL, with the clearance rate reaching 85.2% at the highest tested concentration (8.0 mg/mL), an antioxidant capacity equivalent to 1.46 mM Trolox. The moderate discrepancy between the two IC_50_ values is a common observation for complex plant extracts [29,30]. These IC_50_ values indicate that the UAE-DES extraction coupled with AB-8 resin purification is an effective strategy for recovering antioxidant flavonoids.

### 2.6. Anti-Inflammatory Activity of the Purified H. dulcis Flavonoids

Macrophages are pivotal in the inflammatory response. In addition, previous studies have demonstrated that *H. dulcis* contributes to alleviating hepatic steatosis and inflammation through the modulation of lipid and inflammatory pathways [2]. Therefore, we used LPS-stimulated macrophage model to study the anti-inflammatory activity of purified *H. dulcis* flavonoids. As depicted in Figure 6A, treating RAW 264.7 macrophages with the purified *H. dulcis* flavonoids at concentrations ranging from 0.06 to 0.96 mg/mL in the presence of 0.8 μg/mL LPS for 24 h did not exhibit any significant cytotoxicity. Nitric oxide (NO) is a prominent pro-inflammatory mediator synthesized by inducible nitric oxide synthase (iNOS) during the inflammatory response. The overproduction of NO triggers oxidative stress and exacerbates tissue damage. As illustrated in Figure 6B, purified *H. dulcis* flavonoids significantly reduced NO production in LPS-stimulated RAW264.7 cells at 0.24 mg/mL (*p* < 0.05); at a concentration of 0.48 mg/mL, the release of nitric oxide was significantly reduced (*p* < 0.001). Co-treatment with the purified flavonoid extract significantly and dose-dependently mitigated this LPS-induced NO overproduction.

The potent dose-dependent suppression of LPS-induced NO overproduction by the purified flavonoids underscores their profound immunomodulatory potential. Recent studies on *Hovenia dulcis* extracts established that such anti-inflammatory efficacy is fundamentally governed by the downregulation of pro-inflammatory enzymes via the blockade of intracellular signaling cascades. The active constituents in the extract, such as dihydromyricetin, have been reported to inhibit mitogen-activated protein kinases (MAPKs) and signal transducer and activator of transcription 1 (STAT1) signaling pathways in LPS-stimulated macrophages [31,32], which may theoretically explain the significant reduction in NO production observed in the present study. These results validate that the UAE-DES extracted flavonoids that possess substantial in vitro anti-inflammatory properties, highlighting their immense potential as natural therapeutic agents.

## 3. Materials and Methods

### 3.1. Chemicals and Reagents

Choline chloride, urea, glycerol, 1,4-butanediol, and malonic acid used for DES synthesis were purchased from Macklin Biochemical Technology Co., Ltd. (Shanghai, China). Chemicals utilized for the colorimetric determination of total flavonoids, including sodium nitrite (NaNO_2_), aluminum nitrate (Al(NO_3_)_3_), and sodium hydroxide (NaOH), were supplied by Jizhi Biochemical Technology Co., Ltd. (Shanghai, China). AB-8 macroporous resin used for purification was purchased from Solarbio Science & Technology Co., Ltd. (Beijing, China). Deionized water was used throughout all experiments. All chemical reagents used in this study were of analytical grade.

### 3.2. Plant Materials

The dried fruits of *H. dulcis*. were purchased from Nanjing Tongrentang Pharmaceutical Co., Ltd. (Nanjing, China). The botanical characteristics of the plant material have been formally identified by Dr. Lei Zhou. The voucher specimens (Voucher No. GDPU-2023-HD01) are housed in the School of Pharmacy at Guangdong Pharmaceutical University (Guangzhou, China). To ensure the homogeneity and consistency of the raw materials, a standardized pre-treatment protocol was employed. The *H. dulcis* fruits were mechanically ground and passed through a 60-mesh sieve. The resulting powder was sealed and stored for later use. Prior to conducting the extraction experiments, the powder was dried for 1 h in a constant-temperature oven at 60°C and then stored in a sealed container. To obtain representative experimental samples, the powder was thoroughly shaken and sampled using the standard quartering method.

### 3.3. Preparation of DESs

DESs were prepared using a standard heating and stirring method. Choline chloride (ChCl) was used as the hydrogen-bond acceptor (HBA) and was mixed with four different hydrogen-bond donors (HBDs), including urea, glycerol, butylene glycol, and malonic acid, at specific HBA-to-HBD molar ratios (1:1, 1:2, and 1:3). The components were accurately weighed, placed in a beaker, and then heated in a 90°C water bath while being continuously stirred. This process continued until all solid components had completely dissolved, ultimately forming a uniform, clear, and transparent liquid mixture.

### 3.4. Extraction Process and Optimization of Extraction Parameters

#### 3.4.1. General Ultrasound-Assisted Extraction (UAE) Procedure

An accurately weighed sample of dried *H. dulcis* fruits powder (0.2 g) was thoroughly mixed with a predetermined volume of the selected DES (with a specific water content) in a centrifuge tube. The mixture was then placed into an ultrasonic bath (WM-500DE, Shanghai Weimi Instruments Co., Ltd., China). Extraction was performed under specific temperature, power, and time conditions. After ultrasonic extraction, the suspension was centrifuged at 4500 rpm for 10 min to separate the solid residues. The supernatant was collected, appropriately diluted, and filtered in preparation for subsequent determination of total flavonoid yield.

#### 3.4.2. Single-Factor Experiments

Five single-factor experiments were systematically conducted to identify the optimal ranges for the key extraction parameters. The default extraction conditions were set initially, and the effect of each individual factor on the total flavonoid extraction yield was evaluated by varying one parameter while keeping the others constant. The investigated variables and their respective ranges were as follows: water content in DES (5–100%, *v*/*v*), liquid-to-solid ratio (5:1–100:1 mL/g), extraction temperature (25–65 °C), ultrasonic time (10–60 min), and ultrasonic power (100–500 W). All experiments were conducted in triplicate to ensure reproducibility.

#### 3.4.3. Box–Behnken Design (BBD) Experiment

A three-factor, three-level Box–Behnken design (BBD) combined with response surface methodology (RSM) to systematically optimize the extraction process based on the preliminary outcomes of the single-factor experiments. The three most influential factors—water content (A), liquid-to-solid ratio (B), and extraction time (C)—were selected as independent factors, while the total flavonoid extraction yield served as the response value (Y). The experimental design, mathematical modeling, and variance analysis (ANOVA) were all performed using Design-Expert software (version 12.0, Stat-Ease Inc., Minneapolis, MN, USA). The relationship between the independent variables and the response was fitted to a second-order polynomial equation to navigate the optimal extraction conditions.

### 3.5. Preparation of Standard Curve

A standard stock solution of rutin (B20771, Yuanye Bio-Technology Co., Ltd. Shanghai, China) was prepared, yielding a constant concentration of 2.078 mg/mL. Subsequently, a series of working standard solutions with varying concentrations (0.02078, 0.03118, 0.05197, 0.10394, 0.20788, 0.41576, and 0.83152 mg/mL) were prepared by transferring specific volumes of the stock solution (10, 15, 25, 50, 100, 200, and 400 µL, respectively) into 1.5 mL centrifuge tubes and diluting with 70% ethanol (990, 985, 975, 950, 900, 800, and 600 µL, respectively). For colorimetric development, a 100 μL aliquot of each working standard solution was mixed with 100 μL of a NaNO_2_ solution (50 g/L) and shaken thoroughly. After standing for 6 min at room temperature, 150 μL of an Al(NO_3_)_3_ solution (100 g/L) was added, shaken, and left for another 6 min. Next, 400 μL of a NaOH solution (200 g/L) was added, and the mixture was adjusted to the final volume with 70% ethanol and allowed to stand for 15 min. Using 70% ethanol as a blank group, the absorbance of each reaction mixture was measured at 510 nm. All experiments were performed in triplicate to obtain the average absorbance values. Finally, a standard calibration curve was constructed by plotting the average absorbance values against the corresponding rutin concentrations. The linear regression equation obtained was Y = 6.91033X + 0.08379, with a high correlation coefficient of R^2^ = 0.99979, demonstrating excellent linearity within the tested concentration range.

### 3.6. Determination of the Extraction Yield of Total Flavonoids

A 100 μL aliquot of the appropriately diluted *H. dulcis* extract was transferred into a 1.5 mL centrifuge tube. The colorimetric reaction was carried out strictly following the identical procedure described for the standard curve preparation (Section 3.5), utilizing 100 μL of a NaNO_2_ solution (50 g/L), 150 μL of an Al(NO_3_)_3_ solution (100 g/L), and 400 μL of a NaOH solution (200 g/L), with the same incubation times. Using 70% ethanol as the blank, the absorbance of the sample mixture was measured at 510 nm. The total flavonoid concentration of the sample solution was then calculated based on the established linear regression equation. The extraction yield of total flavonoids was subsequently calculated using the following formula:

$$\mathrm{Extraction}\;\mathrm{Yield}(\%)=\frac{C\;\times \;V\;\times \;N}{W\;\times \;1000}\;\times \;100\%$$

where *C* denotes the total flavonoid concentration in the extract calculated from the calibration curve (mg/mL), *V* is the total volume of the extraction solvent (mL), *N* represents the dilution factor, and *W* represents the exact mass of the raw *H. dulcis* powder used for the extraction (g). The constant 1000 is applied for the unit conversion.

### 3.7. Separation and Enrichment of Flavonoids from DESs

The enrichment and separation of flavonoids from the crude DES extracts were carried out using an AB-8 macroporous resin column. Initially, the packed resin column was pre-treated and thoroughly washed with four column volumes (CVs) of deionized water. Subsequently, a 5 mL aliquot of the crude DES extract was loaded onto the column. To effectively remove the highly polar DES components and other water-soluble impurities, the column was first washed with 2 CVs of deionized water. Following the removal of the solvent, the targeted flavonoids were desorbed by eluting the column with 45% (*v*/*v*) ethanol. The elution process was continued until the eluate became completely colorless, ensuring the complete recovery of the flavonoids. Finally, the collected DES aqueous effluent and the flavonoid-rich ethanolic eluate were separately concentrated under reduced pressure utilizing a rotary evaporator.

### 3.8. Qualitative Analysis of Flavonoids by UPLC-Q-Exactive Orbitrap-HRMS

The enriched total flavonoids obtained using the procedure described in Section 3.7 were diluted 1000-fold with 50% (*v*/*v*) aqueous methanol to a final concentration of 0.1 mg/mL. The solution was then centrifuged at 16,000 rpm for 30 min, and the resulting supernatant was collected for subsequent compositional analysis.

For the identification of flavonoid constituents, a UPLC-Q Exactive Orbitrap-HRMS instrument (Thermo Fisher Scientific, Bremen, Germany) was employed. The chromatographic setup consisted of a Waters ACQUITY UPLC BEH C18 column (100 × 2.1 mm, 1.7 µm), with the flow rate held constant at 0.3 mL/min. The elution solvents were 0.1% formic acid in water (mobile phase A) and 0.1% formic acid in acetonitrile (mobile phase B), which were applied as follows: 0–2.0 min, 5.0% B; 2.0–4.0 min, 5.0 → 25.0% B; 4.0–8.0 min, 25.0 → 35.0% B; 8.0–11.0 min, 35.0 → 50.0% B; 11.0–13.0 min, 50.0 → 95.0% B; 13.0–14.4 min, 95.0% B; 14.4–14.5 min, 95.0 → 2.0% B; and 14.5–16.0 min, 2.0% B. Mass detection was performed in negative ESI mode, scanning from *m*/*z* 70 to 1050. The spray voltage was set to −3000 V, and the ion source temperature was maintained at 450 °C. Nitrogen served as the collision gas, with normalized collision energies programmed at 20%, 40%, and 60%, while the heater and curtain gas pressures were fixed at 50 and 13 a.u., respectively. Subsequent data handling was carried out using Progenesis QI v3.0 (Waters Corporation, Milford, MA, USA), which performed baseline correction, peak detection, integration, retention-time alignment, and spectral matching. This process yielded a comprehensive data matrix containing critical information such as retention times, mass-to-charge ratios (*m*/*z*), and peak intensities. Subsequently, the software was used to identify characteristic peaks by comprehensively matching the acquired MS and MS/MS fragmentation spectra against a custom commercial database (MJBIOTCM, Majorbio Bio-Pharm Technology Co., Ltd. Shanghai, China). The mass error threshold was strictly set to less than 10 ppm to ensure highly accurate structural identification of the flavonoid compounds.

### 3.9. Antioxidant Capability

#### 3.9.1. ABTS Radical Scavenging Assay

The ABTS radical scavenging assay was performed using a commercial kit following the manufacturer’s protocol (R32104, Yuanye Bio-Technology Co., Ltd. Shanghai, China). Briefly, the ABTS working solution was made in advance by reacting ABTS with an oxidant and incubating it in the dark for 12 h. Then, 280 µL of the ABTS working solution was combined with 7 µL of the sample (0.125–8.0 mg/mL) or the Trolox reference (used as the positive control standard). The mixture was kept in the dark for 6 min, after which the absorbance was measured at 405 nm. The scavenging percentage was determined according to Equation (1):

(1) $$\mathrm{ABTS}\;\mathrm{radical}\;\mathrm{scavenging}\;\mathrm{activity}(\%)\;=\;\left[\frac{A_{0}-A_{i}}{A_{0}}\right]\times 100\%\;$$

where *A_i_* represents the absorbance of the sample following the reaction (ABTS working solution + sample), and *A*_0_ is the absorbance of the blank control without the sample (ABTS working solution + deionized water). The half-maximal inhibitory concentration (IC_50_) values were calculated using non-linear regression analysis (a four-parameter logistic dose–response curve) in GraphPad Prism version 10.0 (GraphPad Software, San Diego, CA, USA).

#### 3.9.2. DPPH Scavenging Capability

The DPPH radical scavenging capacity of the purified *H. dulcis* flavonoids was determined via a slightly modified spectrophotometric method. Briefly, a 200 µM DPPH stock solution was obtained by dissolving 7.7 mg of DPPH (D273092, Aladdin Biochemical Technology Co., Ltd. Shanghai, China) in 100 mL of absolute ethanol, which was subsequently diluted to a 100 µM working solution. For the measurement, 3.9 mL of this working solution was mixed with 0.1 mL of the flavonoid sample (0.125–8.0 mg/mL), and the mixture was shaken vigorously, and then incubated at room temperature in the dark for 30 min. Following incubation, the absorbance at 510 nm was recorded against a blank using a UV-Vis spectrophotometer, with each test carried out in triplicate. Trolox served as the positive control. The percentage inhibition was calculated according to Equation (2):

(2) $$\mathrm{DPPH}\;\mathrm{radical}\;\mathrm{scavenging}\;\mathrm{activity}(\%)=\left[1-\frac{A_{i}-A_{j}}{A_{0}}\right]\;\times \;100\%\;$$

where *A_i_* represents the absorbance of the sample reacting with the DPPH solution, *A_j_* represents the absorbance of the sample background (sample + absolute ethanol), and *A*_0_ represents the absorbance of the DPPH control without the sample (absolute ethanol + DPPH solution). The IC_50_ values were calculated using non-linear regression analysis (a four-parameter logistic dose–response curve) in GraphPad Prism version 10.0.

### 3.10. Anti-Inflammatory Capability

#### 3.10.1. Cell Culture

RAW 264.7 murine macrophages were sourced from Procell Life Science & Technology Co., Ltd. (Wuhan, China). These cells were cultured in Dulbecco’s Modified Eagle Medium (DMEM) containing 10% (*v*/*v*) fetal bovine serum (FBS) and 1% (*v*/*v*) penicillin-streptomycin. Incubation was performed in a humidified atmosphere of 5% CO_2_ at 37 °C.

#### 3.10.2. Cell Viability Assay

The MTT assay was employed to examine the cytotoxicity of the purified *H. dulcis* flavonoid fraction against RAW 264.7 cells, aiming to identify non-toxic concentrations for subsequent anti-inflammatory tests. Briefly, macrophages were dispensed into 96-well plates at a seeding density of 2 × 104 cells/well and allowed to attach overnight (24 h) at 37 °C. Following attachment, the culture medium was exchanged with fresh medium containing different concentrations of the flavonoid extract, and the cells were incubated for an additional 24 h. Thereafter, 10 µL of MTT solution was added to each well, and the plates were incubated further under light-protected conditions. The resulting formazan crystals were dissolved, and the optical density was measured at 490 nm using a microplate reader (Molecular Devices, San Jose, CA, USA). Cell viability was then normalized to that of the untreated control group and reported as a percentage.

#### 3.10.3. Determination of Nitric Oxide (NO) Production

The anti-inflammatory activity of the purified flavonoids was evaluated by monitoring their inhibitory effect on nitric oxide (NO) generation in LPS-stimulated RAW 264.7 macrophages using the Griess reaction. Cells were seeded into 96-well plates at a density of 3 × 10^4^ cells/well and allowed to adhere for 24 h. They were then exposed simultaneously to LPS (0.8 μg/mL) and different non-cytotoxic concentrations of the flavonoid extract for a further 24 h. Following this treatment, cell culture supernatants were harvested and combined with an equal volume of Griess reagent (G9440, Solarbio Science & Technology Co., Ltd. Beijing, China), following the manufacturer’s protocol precisely. The absorbance was read at 540 nm using a microplate reader, and NO concentrations in the samples were determined by referencing a standard calibration curve constructed with sodium nitrite (NaNO_2_).

## 4. Conclusions

In this study, an efficient and eco-friendly ultrasound-assisted deep eutectic solvent extraction method coupled with AB-8 macroporous resin purification was successfully developed for the recovery of bioactive flavonoids from *Hovenia dulcis*. Under the BBD-optimized conditions (water content of 36%, liquid-to-solid ratio of 42:1 mL/g, and extraction time of 42 min), a robust and reproducible yield of 9.09 ± 0.04% was achieved. Phytochemical profiling via UPLC-Q-Exactive Orbitrap-HRMS revealed that the enriched extract was predominantly composed of structurally diverse flavonoids. Furthermore, the purified extract demonstrated potent in vitro antioxidant capacities against DPPH and ABTS radicals, alongside remarkable anti-inflammatory efficacy by significantly suppressing LPS-induced NO production in RAW 264.7 macrophages without inducing toxicity. Taken together, these findings confirm that the optimized green extraction strategy effectively preserves the structural and functional integrity of *H. dulcis* flavonoids. This study provides a powerful and sustainable approach for valorizing *H. dulcis* biomass into high-value functional ingredients for future applications in the food, nutraceutical, and pharmaceutical industries. Future studies will focus on applying this optimized green protocol to diverse commercial *H. dulcis* products and exploring scale-up feasibility for industrial applications.

## Figures and Tables

**Figure 1:**
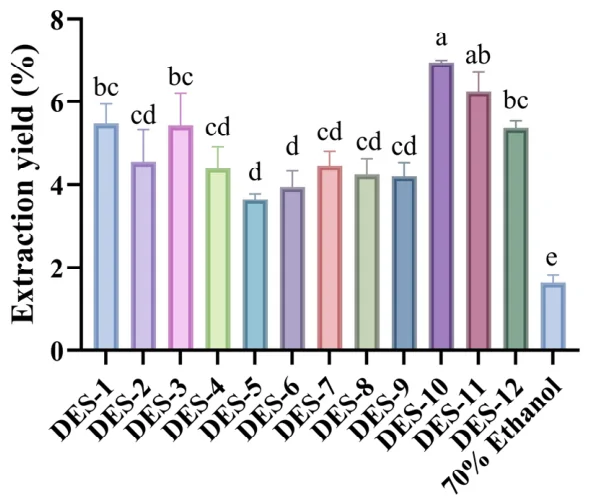
Flavonoid yield from *H. dulcis* obtained by extraction with 12 DESs and 70% ethanol. Data are expressed as mean ± SD (n = 3). Different lowercase letters (a–e) above the bars indicate statistically significant differences (*p* < 0.05), as determined by one-way ANOVA followed by Tukey’s post hoc test.

**Figure 2:**
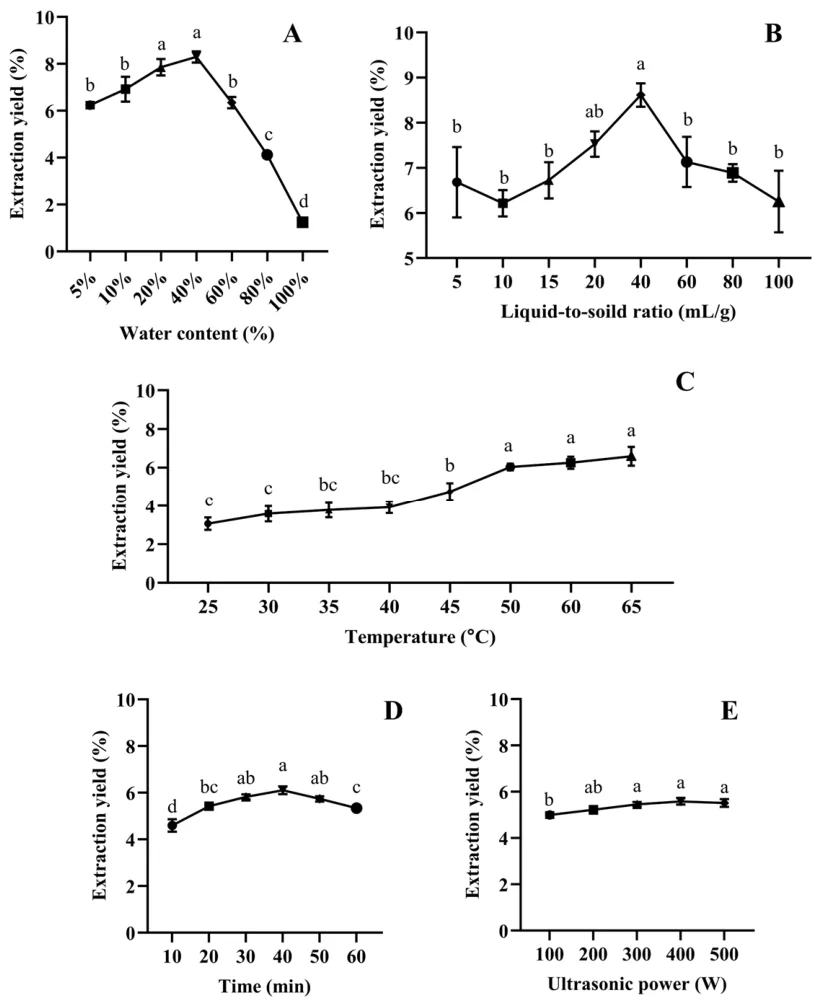
Single-factor experiments to optimize the extraction yield. (**A**) Effect of water content in DES. (**B**) Effect of liquid-to-solid ratio. (**C**) Effect of extraction temperature. (**D**) Effect of ultrasonic extraction time. (**E**) Effect of ultrasonic extraction power. Data are expressed as mean ± SD (n = 3). Different lowercase letters (a–d) adjacent to the data points indicate statistically significant differences (*p* < 0.05), as determined by one-way ANOVA followed by Tukey’s post hoc test.

**Figure 3:**
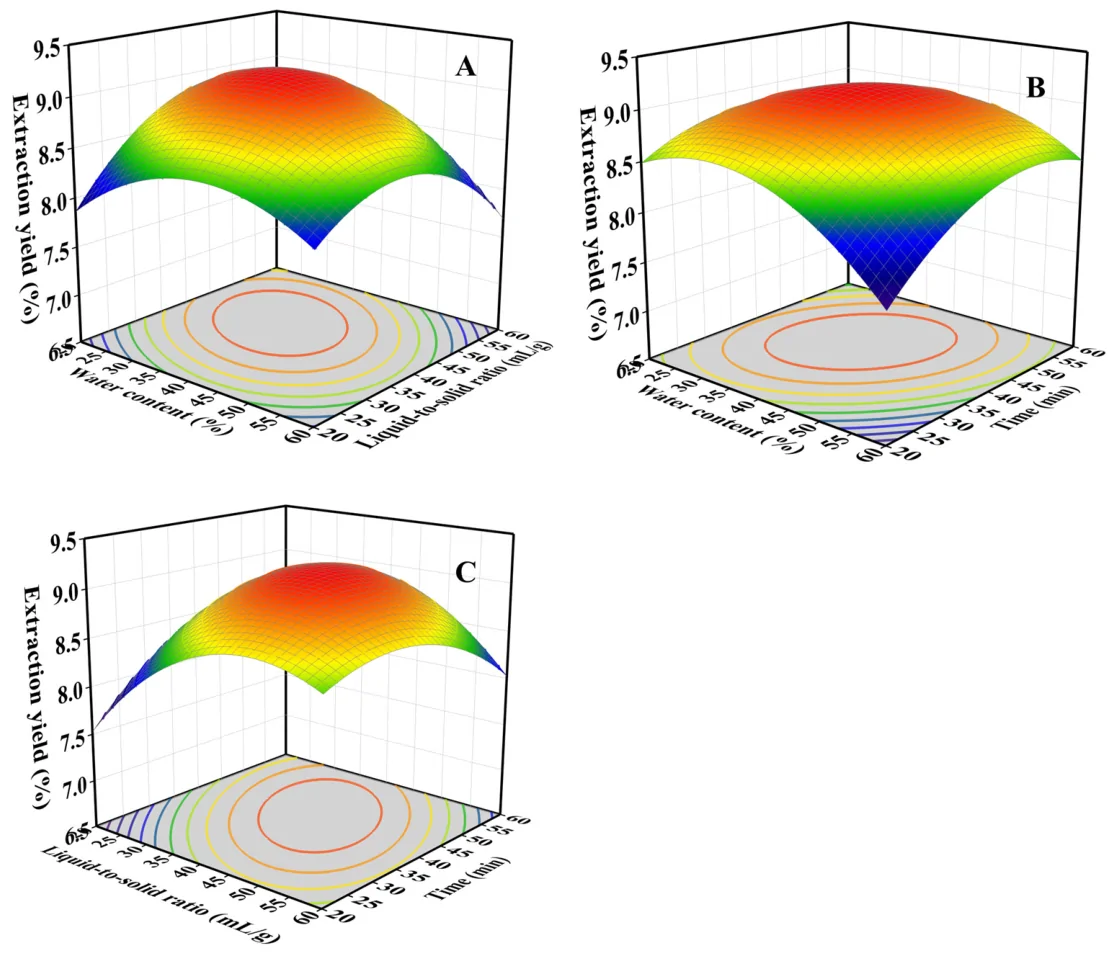
3D response surface curve showing the effects of the independent variables on the flavonoid extraction yield. (**A**) Mutual effects of water content in DES and liquid-to-solid ratio on extraction yield (**A**,**B**). (**A**) Mutual effects of water content in DES and ultrasonic extraction time on extraction yield (**A**,**C**). Mutual effects of liquid-to-solid ratio and ultrasonic extraction time on extraction yield (**B**,**C**).

**Figure 4:**
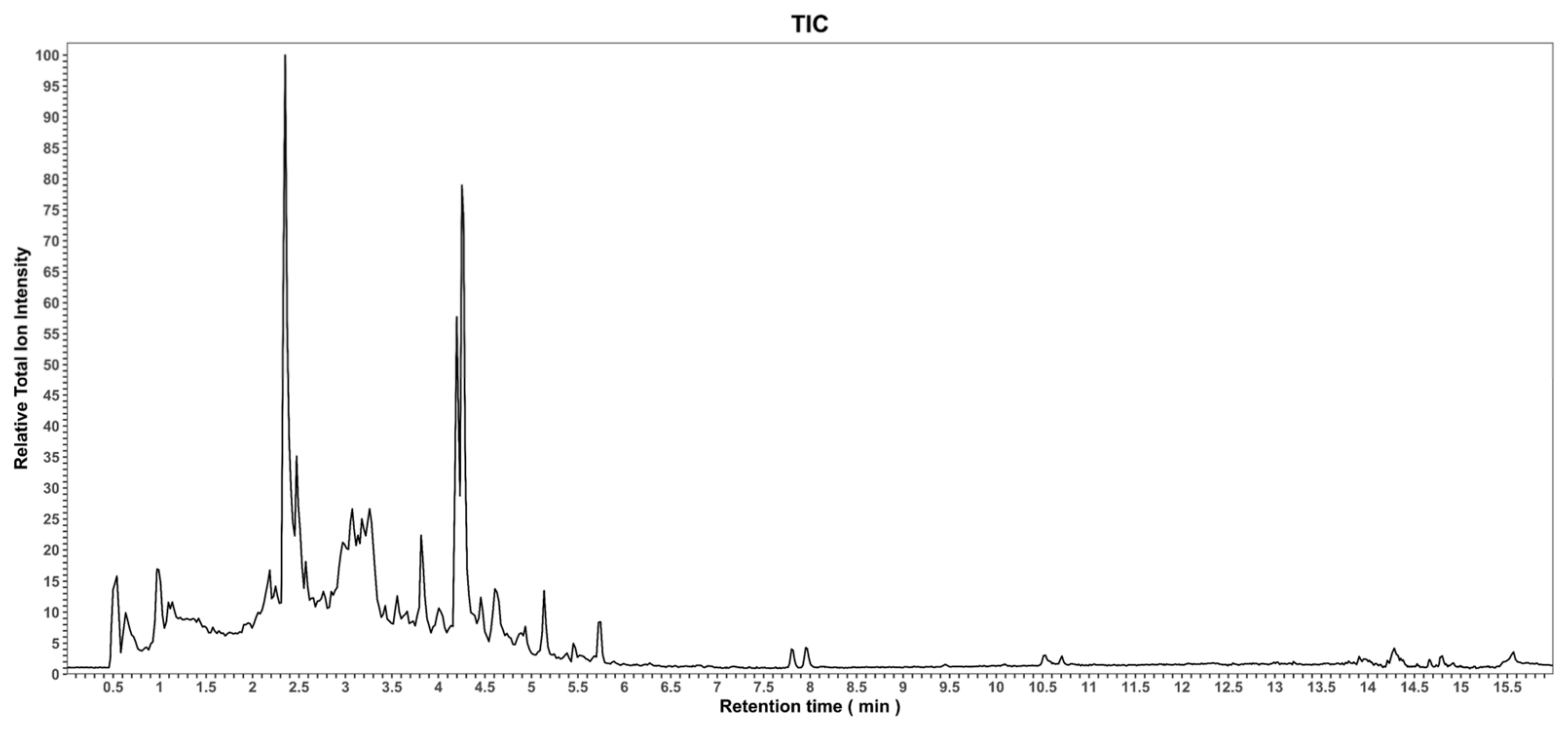
Total Ion Chromatogram of purified *H. dulcis* flavonoids (negative ion mode).

**Figure 5:**
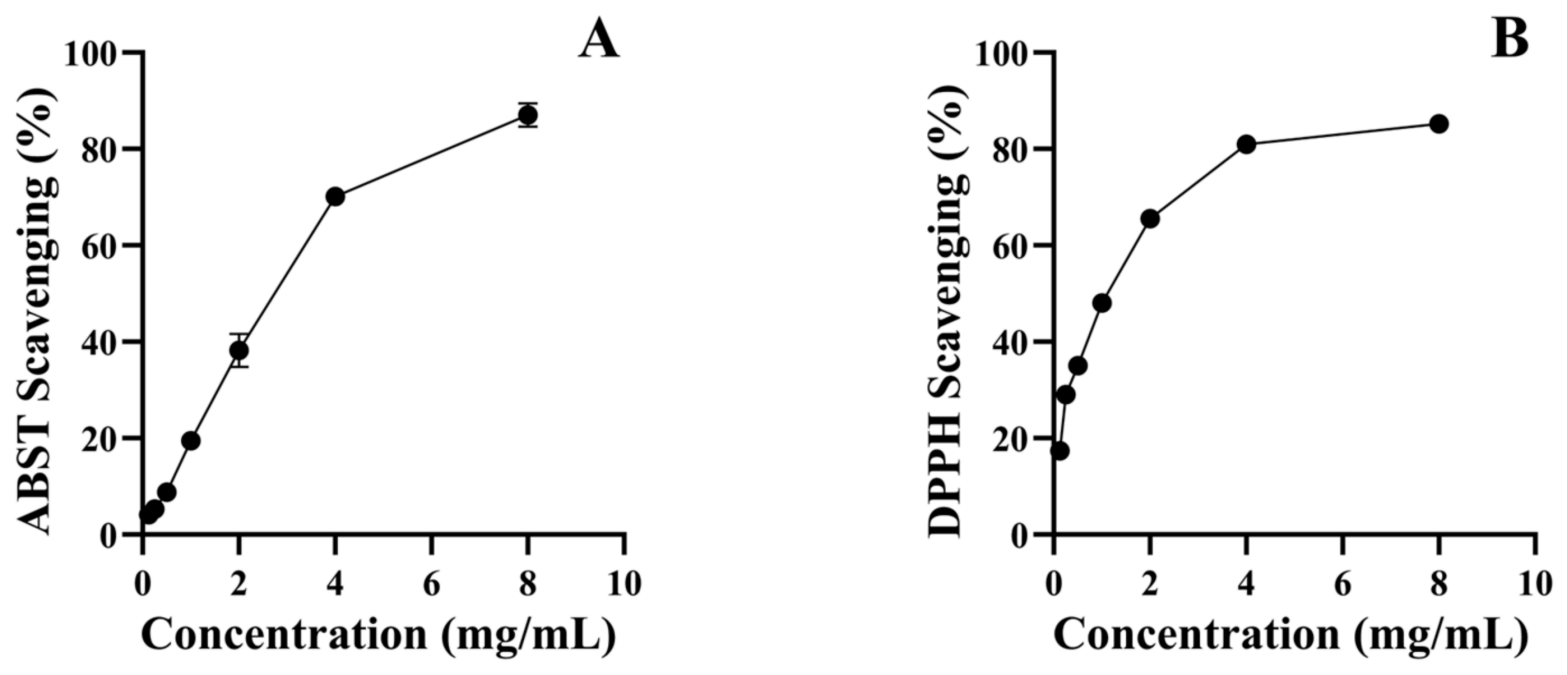
The antioxidant activities of the purified *H. dulcis* flavonoids in vitro. (**A**) ABTS radical scavenging capability. (**B**) DPPH radical scavenging capability. Data are expressed as mean ± SD (n = 3).

**Figure 6:**
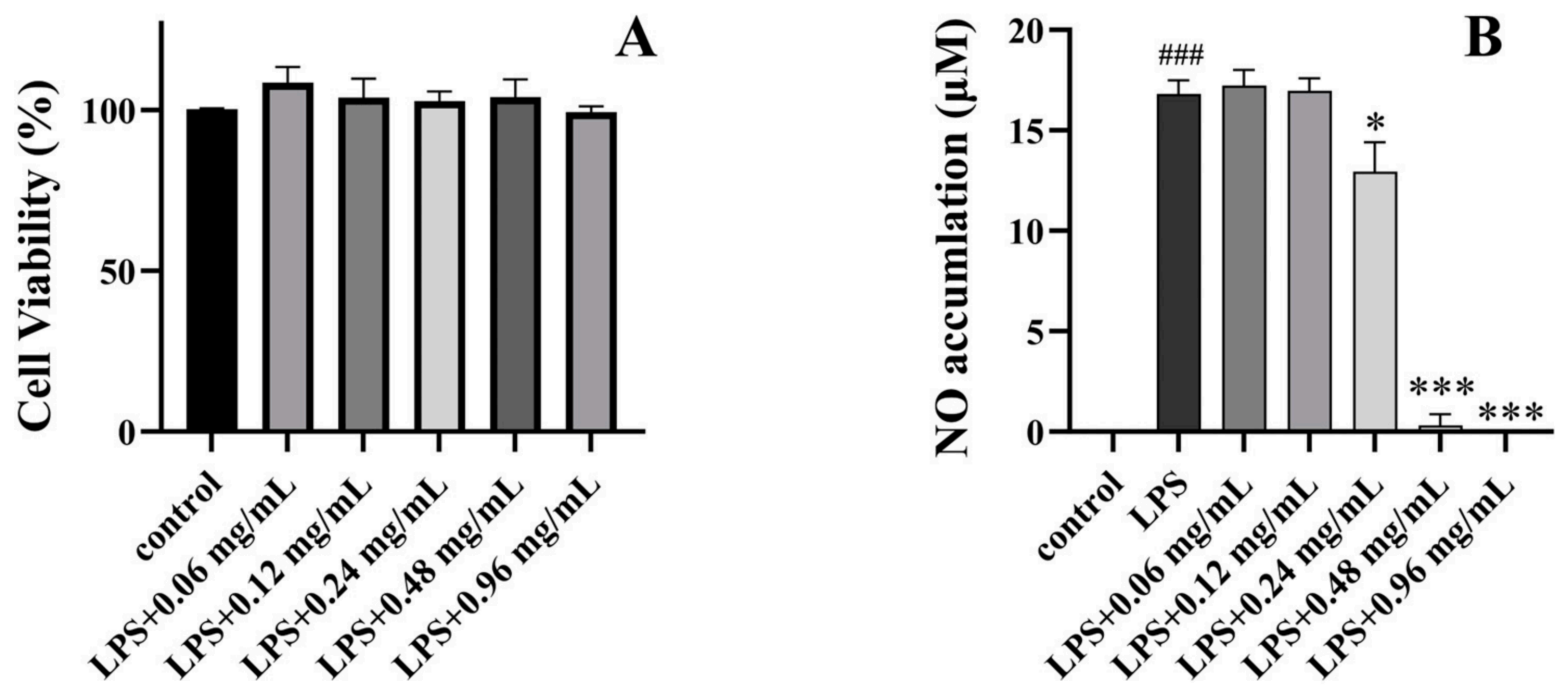
The impact of purified *H. dulcis* flavonoids on the generation of NO by macrophages stimulated by LPS. (**A**) RAW 264.7 cells were treated with the indicated concentrations of purified *H. dulcis* flavonoids in the presence of LPS (0.8 μg/mL) for 24 h. Cell viability was measured using the MTT assay. (**B**) RAW 264.7 cells were treated with the indicated concentrations of purified *H. dulcis* flavonoids in the presence of LPS (0.8 μg/mL) for 24 h. NO production was determined using the Griess reagent. Data are expressed as mean ± SD (n = 3), ### *p* < 0.001 vs. control, * *p* < 0.05, *** *p* < 0.001 vs. LPS.

**Table 1:** Different combinations and ratios of deep eutectic solvents tested.

DES	HBA	HBD	Molar Ratio (HBA:HBD)
DES-1	Choline chloride	Urea	1:1
DES-2	Choline chloride	Urea	1:2
DES-3	Choline chloride	Urea	1:3
DES-4	Choline chloride	Glycerol	1:1
DES-5	Choline chloride	Glycerol	1:2
DES-6	Choline chloride	Glycerol	1:3
DES-7	Choline chloride	1,4-Butanediol	1:1
DES-8	Choline chloride	1,4-Butanediol	1:2
DES-9	Choline chloride	1,4-Butanediol	1:3
DES-10	Choline chloride	Malonic acid	1:1
DES-11	Choline chloride	Malonic acid	1:2
DES-12	Choline chloride	Malonic acid	1:3

**Table 2:** Box–Behnken experimental design with the actual and response values for the extraction yield.

Run	Water Content (%)	Liquid-to-Solid Ratio (mL/g)	Time (min)	Extraction Yield (%)
1	40	40	40	9.07 ± 0.06
2	60	20	40	8.16 ± 0.07
3	40	60	20	8.49 ± 0.06
4	20	20	40	7.82 ± 0.05
5	20	40	60	8.42 ± 0.03
6	40	40	40	9.18 ± 0.02
7	40	20	60	8.62 ± 0.07
8	40	60	60	8.04 ± 0.05
9	40	40	40	9.05 ± 0.04
10	20	60	40	8.61 ± 0.05
11	40	40	40	9.11 ± 0.08
12	20	40	20	8.54 ± 0.07
13	60	60	40	7.73 ± 0.03
14	40	20	20	7.51 ± 0.06
15	60	40	60	8.38 ± 0.07
16	40	40	40	9.12 ± 0.05
17	60	40	20	7.68 ± 0.07

**Table 3:** Regression model analysis of variance.

Source	Sum of Squares	*df*	Mean Square	F-Value	*p*-Value	Significance
Model	4.85	9	0.5386	138.01	<0.0001	***
A	0.2592	1	0.2592	66.41	<0.0001	***
B	0.0722	1	0.0722	18.50	0.0036	**
C	0.1922	1	0.1922	49.25	0.0002	**
AB	0.3721	1	0.3721	95.34	<0.0001	***
AC	0.1681	1	0.1681	43.07	0.0003	**
BC	0.6084	1	0.6084	155.89	<0.0001	***
A2	0.9222	1	0.9222	236.29	<0.0001	***
B2	1.31	1	1.31	335.91	<0.0001	***
C2	0.6176	1	0.6176	158.25	<0.0001	***
Residual	0.0273	7	0.0039			
Lack of fit	0.0172	3	0.0057	2.27	0.2229	n.s.
Pure Error	0.0101	4	0.0025			
Cor Total	4.87	16				
R^2^	0.9944					
Adjusted R^2^	0.9872					
Predicted R^2^	0.9403					

*** *p* < 0.001; ** *p* < 0.01; n.s., not statistically significant (*p* > 0.05).

**Table 4:** Identification of the chemical constituents of purified *H. dulcis* flavonoids by UPLC-Q Exactive-HRMS.

No.	Compound	Quasi-Molecular Weight	t_R_ (min)	Adducts	Formula	Mass Error (ppm)	Classification
1	Dihydromyricetin	319.04656	3.66	[M-H]^−^	C_15_H_12_O_8_	1.93	Flavanonol
2	Dihydroquercetin	303.25218	4.46	[M-H]^−^	C_15_H_12_O_7_	2.42	Flavanonol
3	Myricetin	317.03090	4.89	[M-H]^−^	C_15_H_10_O_8_	1.92	Flavonol
4	Quercetin	301.03609	5.91	[M-H]^−^	C_15_H_10_O_7_	2.37	Flavonol
5	Kaempferol	285.04107	7.64	[M-H]^−^	C_15_H_10_O_6_	2.11	Flavonol
6	Pinobanksin	271.06190	7.91	[M-H]^−^	C_15_H_12_O_5_	2.57	Flavanonol
7	Pinocembrin	255.06658	10.39	[M-H]^−^	C_15_H_12_O_4_	1.18	Flavonone
8	Galangin	269.04598	10.52	[M-H]^−^	C_15_H_10_O_5_	1.61	Flavonol
9	Kaempferide	299.05647	10.77	[M-H]^−^	C_16_H_12_O_6_	1.19	Flavonol
10	Izalpinin	283.06164	11.06	[M-H]^−^	C_16_H_12_O_5_	1.57	Dihydrochalcone
11	Parvisoflavone B	351.08803	12.68	[M-H]^−^	C_20_H_16_O_6_	1.76	Flavone
12	Atalantoflavone	335.09313	12.87	[M-H]^−^	C_20_H_16_O_5_	1.88	Flavone

## Data Availability

Data are contained within the article.

## Supplementary Materials

The following supporting information can be downloaded at: https://www.mdpi.com/article/10.3390/molecules31193425/s1, Supplementary Figure S1: Structures of 12 flavonoids identified from *Hovenia dulcis* Thunb.

## Abbreviations

The following abbreviations are used in this manuscript:

UAE	Ultrasonic-assisted
DES	Deep eutectic solvent
BBD	Box–Behnken design
UPLC-Q-Exactive Orbitrap-HRMS	Ultra-Performance Liquid Chromatography Coupled with Quadrupole-Exactive Orbitrap High-Resolution Mass Spectrometry
DPPH	2,2-Diphenyl-1-picrylhydrazyl
ABTS	2,2′-Azino-bis(3-ethylbenzothiazoline-6-sulfonic acid)
NO	Nitric oxide
LPS	Lipopolysaccharide
HBA	Hydrogen-bond acceptor
HBD	Hydrogen-bond donor
RSM	Response surface methodology
ANOVA	Analysis of variance
CVs	Column volumes
DMEM	Dulbecco’s Modified Eagle Medium
FBS	Fetal bovine serum
MTT	3-(4,5-dimethylthiazol-2-yl)-2,5-diphenyltetrazolium bromide
ChCl	Choline chloride
MA	Malonic acid
C.V.	Coefficient of variation
IC_50_	Half-maximal inhibitory concentration
MAPKs	Mitogen-activated protein kinases
STAT1	Signal transducer and activator of transcription 1

## References

[1] Song G., Kim H.L., Jung Y., Park J., Lee J.H., Ahn K.S., Kwak H.J., Um J.Y. (2020). Fruit of *Hovenia dulcis* Thunb. Induces Nonshivering Thermogenesis through Mitochondrial Biogenesis and Activation by SIRT1 in High-Fat Diet-Fed Obese Mice and Primary Cultured Brown Adipocytes. J. Agric. Food Chem..

[2] Choi R.Y., Woo M.J., Ham J.R., Lee M.K. (2017). Anti-steatotic and anti-inflammatory effects of *Hovenia dulcis* Thunb. extracts in chronic alcohol-fed rats. Biomed. Pharmacother..

[3] de Godoi R.S., Garcia A.L.H., Borges M.S., George H.K., Ferraz A.B.F., Correa D.S., da Silva F.R., da Silva J. (2023). Protective effect of *Hovenia dulcis* Thunb. leaf extracts against ethanol-induced DNA damage in SH-SY5Y cells. J. Ethnopharmacol..

[4] Jegal K.H., Park H.R., Choi B.R., Kim J.K., Ku S.K. (2023). Synergistic Protective Effect of Fermented Schizandrae Fructus Pomace and Hoveniae Semen cum Fructus Extracts Mixture in the Ethanol-Induced Hepatotoxicity. Antioxidants.

[5] Paik D.H., Lee K.W., Shim Y.Y., Reaney M.J.T., Park I., Lee S.H., Park J.Y., Park E., Lee S.B., Kim I.A. (2024). Efficacy of *Hovenia dulcis* Fruit Extract in Hangover Mitigation: Double-Blind Randomized Clinical Evaluation. Foods.

[6] Jiang W., Ding K., Liu X., Hu Z., Yue R., Yang M. (2026). Dihydromyricetin in the management of diabetes and its complications: A narrative review. Food Funct..

[7] Yang B., Luo Y., Sang Y., Kan J. (2022). Isolation, purification, structural characterization, and hypoglycemic activity assessment of polysaccharides from *Hovenia dulcis* (Guai Zao). Int. J. Biol. Macromol..

[8] Zhu J., Deng Y., Zhang X., Zhou X., Sang Y., Yang B. (2025). Quercetin-*Hovenia dulcis* polysaccharide complexes inhibit alpha-glucosidase and mitigate hyperglycemia. Int. J. Biol. Macromol..

[9] Yang J., Shen D., Wu F., Fu T., Liu B., Xu F. (2025). Total Flavonoids of *Hovenia dulcis* Seeds Ameliorate Hepatic Fibrosis via Modulating PI3K/AKT Signaling Pathway In Vitro and In Vivo. Chem. Biodivers..

[10] Choi C.Y., Cho S.S., Yoon I.S. (2018). Hot-water extract of the branches of *Hovenia dulcis* Thunb. (Rhamnaceae) ameliorates low-fiber diet-induced constipation in rats. Drug Des. Dev. Ther..

[11] Zhang Z., Yu A., Hu W., Wu L., Yang D., Fu L., Wang Z., Kuang H., Wang M. (2024). A review on extraction, purification, structural characteristics, biological activities, applications of polysaccharides from *Hovenia dulcis* Thunb. (Guai Zao). Int. J. Biol. Macromol..

[12] Liga S., Paul C., Peter F. (2023). Flavonoids: Overview of Biosynthesis, Biological Activity, and Current Extraction Techniques. Plants.

[13] Yusoff I.M., Mat Taher Z., Rahmat Z., Chua L.S. (2022). A review of ultrasound-assisted extraction for plant bioactive compounds: Phenolics, flavonoids, thymols, saponins and proteins. Food Res. Int..

[14] Pires I.V., da Silva L.H.M., Rodrigues A., Saldana M.D.A. (2024). Natural deep eutectic solvents for anthocyanin extraction from agricultural sources: Process parameters, economic and environmental analysis, and industrial challenges. Compr. Rev. Food Sci. Food Saf..

[15] Li D. (2022). Natural deep eutectic solvents in phytonutrient extraction and other applications. Front. Plant Sci..

[16] Martins K.P., Hernandez Brito N.L., Costa G.B., Silva Ramos J., Silvestre Barbosa Alessi A.C., Santos T.A., Melo da Silva A.E., Machado I.F., Medeiros Marques L.L., Droval Arcain A.A. (2025). Natural Deep Eutectic Solvents (NADES) in Food Systems: Emerging Applications, Extraction Efficiency, Safety Concerns, and Regulatory Challenges. J. Agric. Food Chem..

[17] El Maaiden E., El Kahia H., Nasser B., Moustaid K., Qarah N., Boukcim H., Hirich A., Kouisni L., El Kharrassi Y. (2023). Deep eutectic solvent-ultrasound assisted extraction as a green approach for enhanced extraction of naringenin from Searsia tripartita and retained their bioactivities. Front. Nutr..

[18] Kutlu N., Kamiloglu A., Abca T.E., Yilmaz O. (2024). Ultrasound-assisted deep eutectic solvent extraction of bioactive compounds from persimmon calyx. J. Food Sci..

[19] Iftikhar H., Akram S., Khalid N.U., Ahmed D., Khan M.H., Ashraf R., Mushtaq M. (2025). Deep eutectic solvent-based green extraction of Strychnos potatorum seed phenolics: Process optimization via response surface methodology and artificial neural network. Talanta.

[20] Meng Y., Zou W., Guo T., Zhang L., Zhang P., Shu P. (2025). Optimization of ultrasound-assisted deep eutectic solvent extraction, characterization, and bioactivities of flavonoids from Cercis glabra leaves. Ultrason. Sonochem..

[21] Lai J., Zhou P., Li X., Lu Y., Wang Y., Yuan H., Yang Y. (2025). Ultrasound-assisted deep eutectic solvent extraction of flavonol glycosides from Ginkgo biloba: Optimization of efficiency and mechanism. Ultrason. Sonochem..

[22] Jiao P., He X., Zhou Z., Wang X., Zhang Y., Chang P., Zhang X., Wang P., Yang P., Niu Q. (2025). Enhanced deep eutectic solvent extraction of phenolic compounds from hawthorn seeds with ultrasonic and resin in-situ adsorption assistance: Process optimization, component analysis, and extraction mechanism. Food Chem..

[23] Bajkacz S., Adamek J., Sobska A. (2020). Application of Deep Eutectic Solvents and Ionic Liquids in the Extraction of Catechins from Tea. Molecules.

[24] Cao D., Liu Q., Jing W.Q., Tian H.Y., Yan H.Y., Bi W.T., Jiang Y.L., Chen D.D.Y. (2020). Insight into the Deep Eutectic Solvent Extraction Mechanism of Flavonoids from Natural Plant. ACS Sustain. Chem. Eng..

[25] Yu P., Wang H., Wang X., Bai T., Kang C., Zhou J., Fang L. (2026). Microwave-assisted natural deep eutectic solvents extraction of saponins from the fibrous roots of American ginseng: Process optimization, antioxidant activity, and metabolomics analysis. npj Sci. Food.

[26] Sferrazza G., Brusotti G., Zonfrillo M., Temporini C., Tengattini S., Bononi M., Tateo F., Calleri E., Pierimarchi P. (2021). *Hovenia dulcis* Thumberg: Phytochemistry, Pharmacology, Toxicology and Regulatory Framework for Its Use in the European Union. Molecules.

[27] Li M., Wei Z.C., Zhang F.X., Li H.J. (2025). Phytochemical Analysis of Semen Hoveniae and Its Potential Mechanism Against Alcoholic Liver Disease via an Integrated Approach Combining UHPLC-Q-TOF MS, Network Pharmacology, and Molecular Docking. Rapid Commun. Mass Spectrom..

[28] Park J.S., Kim I.S., Shaheed Ur R., Na C.S., Yoo H.H. (2016). HPLC Determination of Bioactive Flavonoids in *Hovenia dulcis* Fruit Extracts. J. Chromatogr. Sci..

[29] Kongolo Kalemba M.R., Makhuvele R., Njobeh P.B. (2024). Phytochemical screening, antioxidant activity of selected methanolic plant extracts and their detoxification capabilities against AFB(1) toxicity. Heliyon.

[30] Martinez S., Fuentes C., Carballo J. (2022). Antioxidant Activity, Total Phenolic Content and Total Flavonoid Content in Sweet Chestnut (*Castanea sativa* Mill.) Cultivars Grown in Northwest Spain under Different Environmental Conditions. Foods.

[31] Jeong Y.H., Oh Y.C., Cho W.K., Yim N.H., Ma J.Y. (2019). Hoveniae Semen Seu Fructus Ethanol Extract Exhibits Anti-Inflammatory Activity via MAPK, AP-1, and STAT Signaling Pathways in LPS-Stimulated RAW 264.7 and Mouse Peritoneal Macrophages. Mediat. Inflamm..

[32] Sun Z., Lu W., Lin N., Lin H., Zhang J., Ni T., Meng L., Zhang C., Guo H. (2020). Dihydromyricetin alleviates doxorubicin-induced cardiotoxicity by inhibiting NLRP3 inflammasome through activation of SIRT1. Biochem. Pharmacol..

