# Supplementary material for: Ultrasound-Assisted Deep Eutectic Solvent Extraction of Flavonoids from Hovenia dulcis Thunb: Optimization, Phytochemical Profiling, and Antioxidant and Anti-Inflammatory Activity Evaluation
Source: Molecules. 2026 Sep 26;31(19):3425. doi: 10.3390/molecules31193425 (PMC13665725; doi:10.3390/molecules31193425)
Supplement: Supplementary file 1 [file molecules-31-03425-s001.zip › molecules-4543099-supplementary.pdf]

Supplementary Figure S1 : Structures of 12 flavonoids identified from *Hovenia dulcis* Thunb.

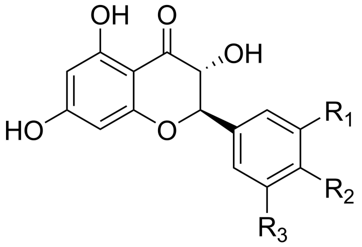

Dihydromyricetin: R_1=OH, R_2=OH,R_3=OH
Dihydroquercetin: R_1=H, R_2=OH,R_3=OH
Pinobanksin:R_1=H, R_2=H, R_3=H

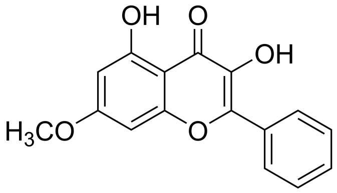

Izalpinin

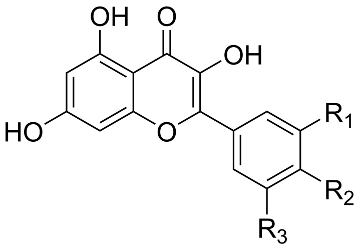

Myricetin: R_1=OH, R_2=OH,R_3=OH
Quercetin: R_1=H, R_2=OH,R_3=OH
Kaempferol: R_1=H, R_2=OH, R_3=H
Galangin: R_1=H, R_2=H, R_3=H
Kaempferide: R_1=H, R_2=OCH_3, R_3=H

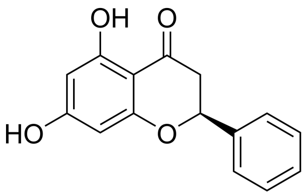

Pinocembrin

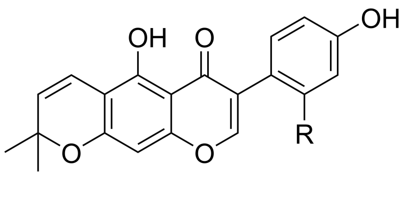

Parvisoflavone B: R=OH
Atalantoflavone: R=H
